# QTL Mapping of the Shape of Type VI Glandular Trichomes in Tomato

**DOI:** 10.3389/fpls.2018.01421

**Published:** 2018-09-26

**Authors:** Stefan Bennewitz, Nick Bergau, Alain Tissier

**Affiliations:** Department of Cell and Metabolic Biology, Leibniz-Institut für Pflanzenbiochemie, Halle, Germany

**Keywords:** tomato, type VI glandular trichomes, trichome morphology, *Solanum habrochaites*, *Solanum lycopersicum*, QTL mapping

## Abstract

Glandular trichomes contribute to the high resistance of wild tomato species against insect pests not only thanks to the metabolites they produce but also because of morphological and developmental features which support the high production of these defense compounds. In *Solanum habrochaites*, type VI trichomes have a distinct spherical shape and a large intercellular storage cavity where metabolites can accumulate and are released upon breaking off of the glandular cells. In contrast, the type VI trichomes of *S. lycopersicum* have a four-leaf clover shape corresponding to the four glandular cells and a small internal cavity with limited capacity for storage of compounds. To better characterize the genetic factors underlying these trichome morphological differences we created a back-cross population of 116 individuals between *S. habrochaites* LA1777 and *S. lycopersicum* var. *cerasiforme* WVa106. A trichome score that reflects the shape of the type VI trichomes allowing the quantification of this trait was designed. The scores were distributed normally across the population, which was mapped with a total of 192 markers. This resulted in the identification of six quantitative trait locus (QTLs) on chromosomes I, VII, VII, and XI. The QTL on chromosome I with the highest LOD score was confirmed and narrowed down to a 500 gene interval in an advanced population derived from one of the back-cross lines. Our results provide the foundation for the genetic dissection of type VI trichome morphology and the introgression of these trichome traits into cultivated tomato lines for increased insect resistance.

**Key Message:** This work shows that the shape of type VI glandular trichomes in tomato is a genetically defined trait controlled by multiple QTLs with one on chromosome I being the major contributor.

## Introduction

Trichomes are differentiations of the epidermis that occur at the surface of the aerial parts of many higher plants. Although they come in a great variety of shapes, they can be classified as either non-glandular or glandular. Glandular (or secreting) trichomes typically have one to a few secretory cells located at the tip of a uni- or multicellular stalk. Glandular trichomes are present in about 30% of all vascular plants (Fahn, 2000). These structures accumulate large quantities of secondary metabolites involved in the protection against herbivores and pathogens (Glas et al., 2012; Tissier, 2012), but also are the source of industrially important ingredients in the flavor, fragrance and pharmaceutical areas (Schilmiller et al., 2008). In most cases, biosynthesis of the compounds takes place in the glandular cells, a feature that is typically correlated with high and specific expression of the pathway enzymes. This has greatly facilitated the identification of the genes involved in the biosynthesis pathways, in particular with the help of trichome specific Express Sequence Tag (EST) and more recently next generation sequencing transcriptome databases. Thus, the biosynthesis of menthol in mint (Croteau et al., 2005), of artemisin in *Artemisia annua* (Brown, 2010), of various terpenoids (Sallaud et al., 2009; Schilmiller et al., 2009) as well as acyl-sugars in tomato (Fan et al., 2016; Schilmiller et al., 2016) or diterpenes in tobacco or rosemary (Sallaud et al., 2012; Scheler et al., 2016) and monoterpenes in thyme (Majdi et al., 2017) could be completely or partially elucidated. In contrast to the progress made in understanding trichome-specific biosynthesis pathways, very little is known about the genes controlling the development and differentiation of glandular trichomes. Much work has been done on the development of non-glandular trichomes in *Arabidopsis thaliana* with numerous transcription factors and regulators identified [reviewed by (Pattanaik et al., 2014)]. However, there is indication that the regulatory mechanisms controlling glandular trichome development may be different for non-glandular trichomes (Serna and Martin, 2006), although initiation mechanisms are likely to be shared (Maes et al., 2011).

Tomato is a major vegetable crop species with over 170 million tons harvested worldwide in 2014, doubling the yield of 1994 (FAOSTAT, 2017). Plants of the Solanum genus possess different types of both glandular and non-glandular trichomes but the abundance and composition of these organs is very diverse (Luckwill, 1943; Channarayappa et al., 1992). The type I and type IV glandular trichomes typically have a single gland cell on a stalk and they are the major site of biosynthesis for acyl sugars (McDowell et al., 2011). Type VII trichomes have a single-celled stalk and a multicellular berry-shaped head and do not seem to be involved in the biosynthesis of secondary metabolites. The type VI is the most prevalent type of glandular trichomes and is found in almost all tomato species (reviewed by Glas et al., 2012). This specific type of trichomes has a four-celled head producing a variety of metabolites including terpenoids or methylketones (in *S. habrochaites* ssp. *glabratum*) but the amount and composition of these metabolites is highly variable in different species and subspecies. The main components in *S. habrochaites* LA1777 are the sesquiterpenes santalene and bergamotene and their carboxylic acids (Coates et al., 1988), both of which are absent in the cultivated tomato *Solanum lycopersicum*. In addition to these distinct chemical contents, there are obvious morphological differences between the type VI trichomes of *S. habrochaites* and *S. lycopersicum*. While the head of the wild tomato species forms a round sphere, the cultivated tomato head looks like a four-leaf clover (Ben-Israel et al., 2009). Furthermore, observations of the type VI trichomes under the microscope show that there is a large intercellular cavity in the trichome head of the wild tomato, whereas this cavity is absent or much smaller in the cultivated tomato (Bergau et al., 2015). This morphological difference is important because it allows large quantities of secondary metabolites to accumulate in the wild species, and thereby contribute to an increased resistance against pests. In a previous study, Ben-Israel et al. (2009) showed a correlation between the expression of secondary metabolite pathway genes and the shape of the trichomes, suggesting that this intercellular storage compartment could arise due to the high flux of metabolites. Recently we could show that the total amount of terpenoids per trichome is 97 fold lower in the *S. lycopersicum* accession LA4024 compared to LA1777 which can be explained at least in part by the near absence of the intercellular cavity in the cultivated tomato (Balcke et al., 2017). However, the genes responsible for this morphological character are still unknown. Because this compartment is extracellular, potential candidates are cell wall modifying genes such as xyloglucan endotransglycosylases/hydrolases (Van Sandt et al., 2007) or pectin methylesterases (Micheli, 2001), components of the cytoskeleton such as microtubules (Lloyd, 2011) or regulating transcription factors like the *Arabidopsis thaliana* MYB5 (Li et al., 2009). Three mutants affecting type VI trichome development in tomato have been described so far, hairless (*hl*), chalcone isomerase (*chi*) and odorless-2 (*od-2*), but these do not seem to affect the development of the storage cavity (Kang et al., 2010, 2014, 2016). The *hl* mutant is affected in a component of the WAVE complex controlling actin nucleation, and thereby supports a link of the cytoskeleton with trichome morphology and terpenoid production. The *chi* mutant has no major morphological defects except a smaller size of type VI trichomes that produce severely reduced levels of terpenes, indicating a metabolic connection between flavonoid and terpenoid metabolisms. The gene responsible for the *od-2* mutation has not been identified to date.

A quantitative trait locus (QTL) is a region on chromosome that can be linked to a particular phenotype (Miles and Wayne, 2008). QTL analyses have been successfully used to map various trichome traits such as trichome specific acyl-sugars (Leckie et al., 2012) as well as type IV trichome density in *S. habrochaites* (Momotaz et al., 2010) and the presence of type IV trichomes in *S. galapagense* (Firdaus et al., 2013).

To investigate the genetic loci underlying the morphological differences in the type VI trichomes between *S. habrochaites* and *S. lycopersicum*, we created a backcross population using *S. lycopersicum* as the recurrent parent. This population was genetically mapped using High-Resolution Melt Analysis (HRM) (Wittwer et al., 2003) and other types of molecular markers. A score was designed to quantify the shape of the type VI trichome heads and used to perform a QTL analysis. Our results indicate that there are multiple loci, with one on chromosome I being the major factor, contributing to this distinct trichome morphology.

## Materials and Methods

### Plant Material and Growth Conditions

Seeds of the tomato accessions were obtained from the Tomato Genetics Resource Center, UC Davis, United States. Seeds of *S. lycopersicum* var. *cerasiforme* ‘West Virginia 106’ (WVa 106) and *S. lycopersicum* var. Ferum were provided by Mathilde Causse (INRA, France). Seeds of the other tomato accessions were obtained from the Tomato Genetics Resource Center, UC Davis, United States. Plants were grown under 16:8 h long day conditions in a climate-controlled greenhouse at 21–24°C and 65% humidity.

### Backcross Population

*Solanum habrochaites* LA1777 and *S. lycopersicum* var. *cerasiforme* ‘West Virginia 106’ (WVa 106) were crossed by using the pollen of LA1777 to manually fertilize emasculated flowers of WVa106. The backcross population was then created by taking the pollen of F1 plants to manually fertilize an emasculated flower of WVa106. The backcross plants were numbered according to the F1 plant from which they originated. For example, plant 4-06 is the sixth backcross plant bred from F1 plant number 4.

Successful backcrosses were confirmed by determining the trichome score (see below), analyzing leaf surface GC-MS profiles and preliminary genotyping for six independent loci.

Two advanced backcross lines were created by self-pollination of the BC1 plants 4-06 and 2-11.

### Trichome Score

**FIGURE 1:**
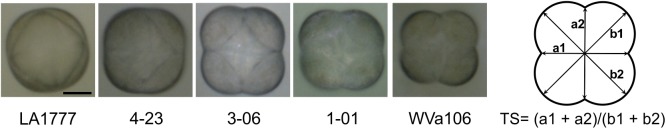
The type VI trichome heads of the parental lines LA1777 and WVa 106 as well as representative backcross lines with intermediate scores as observed with a bright field microscope. Scale bar is 20 μm. The right panel shows the formula to calculate the trichome score (TS).

Type VI glandular trichome heads were imaged with a Nikon AZ100 stereoscope and the trichome diameters were measured using Adobe Photoshop. The trichome score is defined as the ratio of the sum of the diameters through the constriction sites to the sum of the diameters through the wide edges (**Figure 1**). Six to twelve trichomes were scored for each line. A score of 1 would imply a perfectly circular shape.

### Confocal Microscopy of Type VI Trichomes

Microscopy analysis of type VI trichomes was performed on intact leaves with a LSM 780 (Zeiss, Jena, Germany) confocal microscope. Autofluorescence was excited at 405 nm and recorded at 420–545 nm (to visualize the cytoplasm and cell wall) and 645–735 nm (for chlorophyll). The trichomes were analyzed from the top and the images were taken from the middle part of the trichome. The areas of the secretory cavities and the entire trichomes as well as the different diameters were measured by ImageJ^1^ (Fiji, 32 bit).

### GC–MS Analysis of Hexane Surface Extracts

Surface extracts were collected from 6 leaflets of circa 5 cm length by adding 2 ml n-hexane and shaking for 1 min. The hexane was taken off and centrifuged at 16,000 *g* for 90 s to remove debris. 1 μl of the supernatant was injected directly into GC–MS for analysis on a Trace GC Ultra gas chromatograph coupled to an ISQ mass spectrometer (Thermo Scientific) as previously described (Brückner et al., 2014).

### Mapping

Genomic DNA was isolated from 50 to 100 mg fresh leaf material using the Qiagen DNeasy Plant kit and 10 ng DNA was used for each mapping point.

The backcross lines were genotyped for 192 markers by a combination of different techniques. Several markers were taken from the literature (Dal Cin et al., 2009) but most markers were designed utilizing single nucleotide polymorphisms (SNPs) between the parental lines using the tomato genome (Tomato-Genome-Consortium, 2012) for the cultivated tomato and the Solanum trichome EST library (McDowell et al., 2011) as well as our own unpublished RNA sequencing data for LA1777. The markers were chosen to cover the whole tomato genome and aligned to the published tomato genome version SL2.40 annotation ITAG2.3 by José M Jiménez-Gómez, Max Planck Institute for Plant Breeding Research, Cologne, Germany.

Oligonucleotide primers were designed using primer3 (Rozen and Skaletsky, 2000) based tools and synthesized by Eurofins Genomics (Ebersberg, Germany).

### High-Resolution-Melt Analysis

High-resolution melt curves were obtained for 119 markers in a 96 well format using my-Budget 5x EvaGreen^®^ QPCR Mix II (Bio&Sell, Feucht, Germany) on a Bio-Rad Connect 96x Real-Time PCR system. Data analysis was performed using the Bio-Rad Precision Melt Analysis Software V1.1.

### Sequenom iPlex SNP Genotyping

For 66 markers an iPlex Gold multiplex genotyping assay was performed by ATLAS Biolabs (Berlin, Germany).

### Cleaved Amplified Polymorphic Sequences (CAPS)

To analyze the 7 CAPS marker a PCR with specific primers was performed using DreamTaq DNA polymerase (Thermo Scientific) followed by a digest with the respective restriction enzyme (NEB) and separation on an agarose gel.

### QTL Mapping

QTL analysis was performed by composite interval mapping (CIM) using the Windows QTL Cartographer software V2.5 (Wang et al., 2012), at 1 cM walk speed and with a 10 cM window size. Following the guidelines by (Lander and Kruglyak, 1995) a logarithm of odds (LOD) threshold of 2.5, calculated from 1000 permutations at a probability of 0.05, was used for declaring significant QTLs.

Epistatic interactions were determined by using the QTLNetwork software version 2.1 (Yang et al., 2008). Window size, working speed and filtration window for the genome scan were set at 10, 1, and 10 cM, respectively. The *F*-test using Henderson method III was used to determine significance with 1,000 permutations.

## Results

### Designing a Score for Type VI Trichome Shape

**FIGURE 2:**
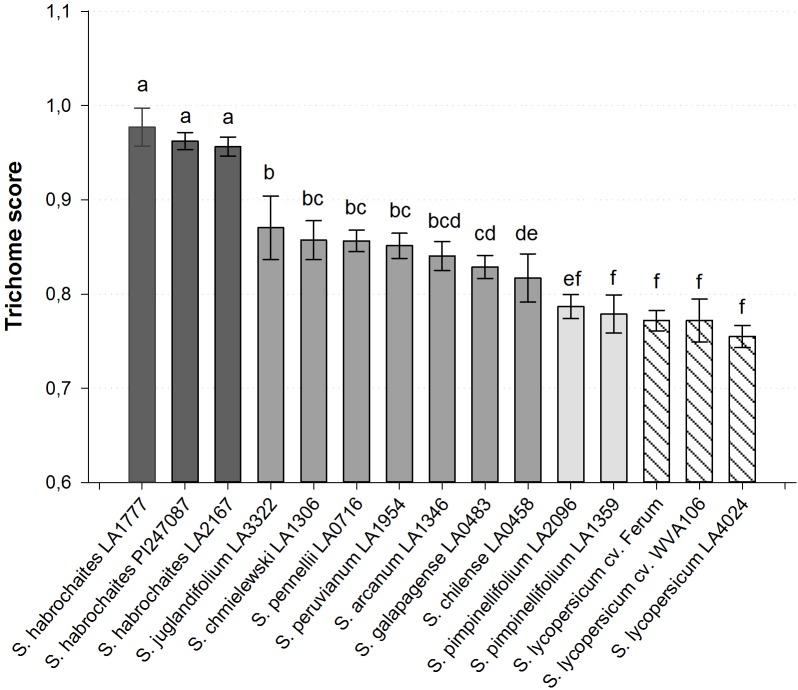
Quantified type VI trichome shape of various wild and cultivated tomato accessions. The trichome score was calculated with standard deviation for *n* ≥ 10 measured trichomes. All *Solanum habrochaites* accessions show a score greater 0.95, *S. lycopersicum* and *S. pimpinellifolium* accession accessions have a score of 0.76–0.79 and all other wild species score between 0.82 and 0.87. Bars with no common letters above them indicate a significantly different score (*p* < 0.001; one-way ANOVA, Tukey pairwise test).

When seen from above, the shape of type VI trichomes in the cultivated tomato (*S. lycopersicum*) and in the wild species *S. habrochaites* can be clearly differentiated (**Figure 1**). The type VI trichomes of *S. habrochaites* have the shape of a ball, whereas in *S. lycopersicum* the four glandular cells can be clearly distinguished giving the head of the trichome the appearance of a four-leaf clover. Observations of harvested type VI trichomes under a fluorescence microscope confirm the presence of four cells and of a large extracellular cavity between the four glandular cells in *S. habrochaites* (**Supplementary Figure S1**). To quantify this difference in the shape of the trichomes, we designed a score TS = (a1 + a2)/(b1 + b2) where a1 and a2 represent the two shortest diameters and b1 and b2 the two longest diameters of the trichome viewed from above. For *S. habrochaites* this score approaches 1 (0.972 ± 0.004), reflecting its almost perfect circular shape, whereas the *S. lycopersicum* score is 0.780 ± 0.006. We calculated this score for a number of different tomato species and cultivars (**Figure 2**). This revealed that all three different *S. habrochaites* lines have the highest score, whereas cultivars of *S. lycopersicum* and *S. pimpinellifolium* have the lowest score. Other wild species like *S. pennellii*, *S. chmielewskii* or *S. peruvianum* have scores that are intermediate and are statistically significantly distinguishable from both *S. habrochaites* and *S. lycopersicum* correlating with their slightly rounder shape (**Figure 2** and **Supplementary Figure S2**).

### Phenotyping a *S. habrochaites* × *S. lycopersicum* Backcross Population for Trichome Traits

In order to investigate the shape of type VI glandular trichomes in wild and cultivated tomatoes we created a backcross population (BC1) by crossing the wild *S. habrochaites* species LA1777 to the cultivated *S. lycopersicum* var. *cerasiforme* WVa106 tomato line, which was used as the recurrent parent. A total of 116 independent backcross lines were identified and confirmed by determining the trichome score, analyzing GC-MS profiles and preliminary genotyping for six independent loci.

**FIGURE 3:**
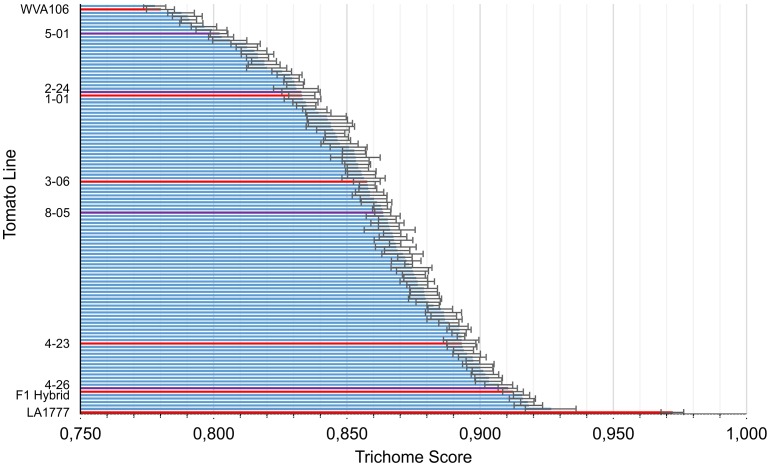
The trichome scores across the backcross population. The parental lines (WVa106 and LA1777) are located at the far ends of the scale. For each line, 6–12 trichome heads were scored. Backcross lines that are shown in **Figure 1** and the F1 hybrid are highlighted in red. Backcross lines that are shown in **Supplementary Figure S3** are highlighted in purple.

The population shows a great diversity in the shape of the type VI glandular trichomes, which we could quantify by calculating the trichome score. The wild tomato LA1777 reaches a score of 0.972 with a standard derivation of 0.018 and the cultivated tomato scores 0.780 ± 0.022. While no backcross line reached scores as high as the wild parent, the highest ones scored about 0.92, around the same as the F1 crosses (0.912 ± 0.019), indicating that the morphology of *S. habrochaites* trichomes is a dominant trait. A few lines showed a phenotype comparable to the cultivated line but the great majority falls in-between the parents, forming a homogeneous gradient (**Figure 3**). According to the Anderson–Darling test the scores followed a normal distribution with a *p*-value of 0.594. To test whether the trichome score reflects the size of the internal cavity we selected four backcross lines with distinct trichome scores and estimated the size of their storage cavity, along with those of the two parental lines and an additional cultivar of tomato (*S. lycopersicum* LA4024). This was done by fluorescence microscopy, which allows us to clearly distinguish the cavity from the cells, by taking pictures where the area of the cavity is the largest. The area of the cavity expressed as percentage of the total area of the trichome head correlates well with the trichome score using a polynomial regression of the second degree (*r*^2^ = 0.93) (**Supplementary Figure S3**). Thus, the trichome score can be considered as a proxy for the size of the intercellular storage cavity of type VI trichomes. The backcross population was screened for the volatile composition. 42 of the 116 lines accumulated the LA1777 specific sesquiterpenes santalene and bergamotene (Sallaud et al., 2009), while the remaining 74 did not. When determining the trichome score of the two subsets, the population lines accumulating santalene scored slightly higher (0.872 ± 0.032) than the other lines (0.853 ± 0.032). A *t*-test shows that the difference is significant with *p* < 0.01.

### Mapping

To perform a QTL analysis to identify genetic regions contributing to the trichome head shape the backcross population was genotyped. This was achieved mainly by using High Resolution Melt analysis (HRM), a technique established to genotype plants (Li et al., 2010) and we supplemented the mapping with CAPS markers and iPLEX sequencing^TM^. This resulted in 192 markers (see complete list in **Supplementary Table S1**), which were plotted onto the ITAG2.4 version of the published tomato map (Tomato-Genome-Consortium, 2012). The whole genome is covered with at least 12 markers per chromosome, a maximum marker distance of 14.7 cM and an average of 5.2 cM. The map spans 933 cM in total. The mapping data is provided in **Supplementary Table S2** and a graphic representation of the linkage map in **Supplementary Figure S4**. We note here that there is currently no complete and annotated genome assembly of *S. habrochaites*, and that the available genomic sequences of *S. habrochaites* have been aligned onto the reference genome of *S. lycopersicum* (Aflitos et al., 2014; Lin et al., 2014). Therefore, although there is overall good synteny between *S. habrochaites* and *S. lycopersicum* at least based on the observation of synaptonemal complexes (Anderson et al., 2010) and fluorescence *in situ* hybridization of bacterial artificial chromosomes (BAC-FISH) (Szinay et al., 2012), small chromosome rearrangements are possible. For example at the virus resistance Ty2 locus which comes from *S. habrochaites* an inversion was detected (Wolters et al., 2015). Thus, we cannot exclude that in the intervals between our markers, the gene order may not be the same.

**FIGURE 4:**
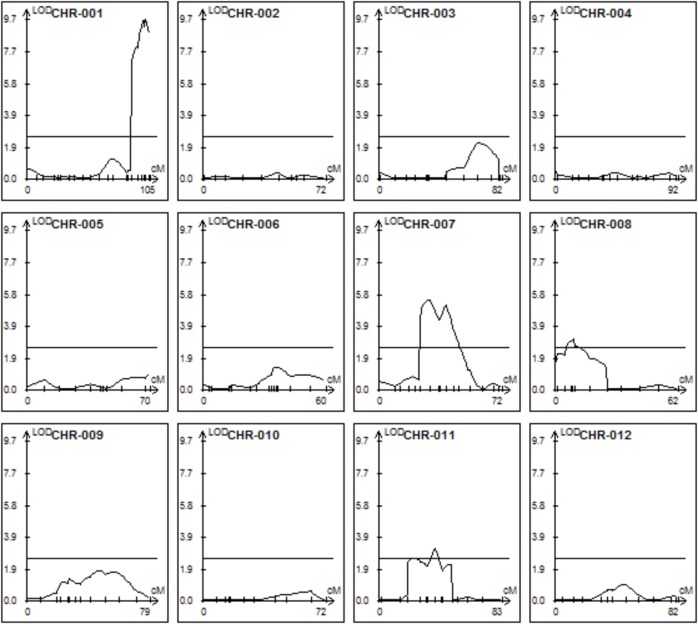
Quantitative trait locus (QTLs) determining trichome shape on the 12 tomato chromosomes. CIM mapping with a LOD threshold of 2.5.

The trichome scores were used in combination with the mapping data to perform a QTL analysis with the Windows QTL Cartographer software V2.5 using composite interval mapping (CIM) algorithm. This revealed six QTLs on four different chromosomes (**Figure 4** and **Table 1**).

**Table 1:** Quantitative trait locus (QTLs) supporting trichome head shape identified using CIM mapping with a LOD threshold of 2.5.

QTL	Chromosome	LOD score	QTL size in cM	Marker closed to the left border	Marker closest to the right border
QTL1	1	9.4	12.7	Solyc01g107130	Solyc01g113620
QTL7a	7	5.5	11.4	Solyc07g053060	Solyc07g054310
QTL7b	7	5.2	10.5	Solyc07g054310	Solyc07g062530
QTL8	8	2.9	4.6	Solyc08g008610	Solyc08g008610
QTL11a	11	2.6	4.2	Solyc11g007580	Solyc11g008860
QTL11b	11	2.9	5.4	Solyc11g010850	Solyc11g012110

All QTLs provide an additive effect with the strong QTL on chromosome I being by far the strongest one and thus the major contributor. In order to see if epistatic interactions underlie the trichome shape we used the QTLNetwork software version 2.1. No significant epistasis could be found.

### Phenotyping and Mapping the Progeny of Selected BC Plants

To support the claim that a QTL on Chromosome I is the main contributor to the trichome shape we looked at a number of self-pollinated backcross lines. Line 4-06 showed one of the highest trichome scores with a value of 0.896 ± 0.011 and was heterozygous for markers covering the associated QTLs on chromosomes I and VII. The 98 individual self-pollinated progeny of 4-06 shows again a segregating trichome score distribution comparable to the backcross population itself with some lines scoring at the cultivated level and some lines around 0.9 but no line reaching wild type levels. When genotyping the QTL on chromosome I we found that all of the 40 selfed plants with the highest trichome scores carry at least one copy of the wild DNA at the QTL on chromosome I while none of the lowest scoring lines is homozygous for LA1777 and 8 of the 10 the lowest scoring lines are homozygous cultivated. The nine top scoring lines are all homozygous wild for at least one of the two strongest QTLs on chromosome I or VII. The average trichome score for the lines homozygous for the *S. habrochaites* markers within the QTL on Chromosome I is 0.873 ± 0.024, significantly higher (*t*-test: *p*-value < 0.05) than the heterozygous (0.856 ± 0.025) or homozygous cultivated lines (0.829 ± 0.023). Unfortunately, no novel recombination event could be detected in the QTL regions on either chromosome I or VII.

In contrast, the backcross lines 2–11 had a low trichome score of 0.831 ± 0.020 and was homozygous for *S. lycopersicum* markers over the QTL on chromosome I. Individuals of the self-pollinated progeny (*n* = 67) do not have higher trichome scores, with an average TS of 0.828 ± 0.028.

### Prospective Candidate Genes in the Identified QTLs

The major QTL on chromosome I spans a genetic interval of 12.7 cM in a relatively gene rich region containing 370 genes (see **Supplementary Table S3**). Assuming that genes responsible for the different shape of type VI trichomes in *S. habrochaites* would be likely to be on the one hand more strongly expressed in trichomes and on the other hand more strongly expressed in LA1777 compared to LA4024, a reduced list of potential candidate genes can be established (see **Table 2**). The 22 genes thus selected encode proteins with quite diverse functional classes, including four unknown proteins, three transcription factors, two cell wall metabolism enzymes, and four metabolic enzymes.

**Table 2:** Potential candidate genes from the trichome shape QTL on chromosome I.

Solyc ID	Annotation	LA1777 leaves	LA1777 trich.	LA4024 leaves	LA4024 trich.	Leaves/trich. LA1777^a^	Leaves/trich. LA4024^a^	1777/4024^b^
01g108530	Acetyl esterase	**30,5**	**241,1**	**35,1**	**19,5**	−3,0	0,8	3,6
01g108550	Unknown Protein	**10,5**	**76,8**	**4,8**	**7,2**	−2,9	−0,6	3,4
01g111950	Receptor-like kinase	**13,7**	**100,3**	**14,8**	**15,0**	−2,9	0,0	2,7
01g110340	Endoglucanase 1	**27,2**	**156,9**	**42,5**	**105,6**	−2,5	−1,3	0,6
01g111350	Nodulin family protein	**143,2**	**685,8**	**24,1**	**63,8**	−2,3	−1,4	3,4
01g108670	Kinesin-like protein	**7,1**	**32,3**	**8,1**	**8,6**	−2,2	−0,1	1,9
01g108580	Gibberellin receptor GID1L2	**18,0**	**74,2**	**57,2**	**32,8**	−2,0	0,8	1,2
01g108100	Unknown Protein	**15,6**	**60,7**	**8,3**	**10,6**	−2,0	−0,3	2,5
01g107390	Auxin-responsive GH3 product	**15,2**	**58,7**	**8,3**	**28,0**	−1,9	−1,7	1,1
01g110290	Squalene synthase	**400,3**	**1274,7**	**648,5**	**450,7**	−1,7	0,5	1,5
01g107920	Os07g0175100 protein	**9,9**	**28,5**	**8,2**	**14,2**	−1,5	−0,8	1,0
01g109300	4-hydroxy-3-methylbut-2-enyl diphosphate reductase	**667,2**	**1834,1**	**863,1**	**1109,8**	−1,5	−0,4	0,7
01g109980	BEL1-like homeodomain protein 6	**87,9**	**234,6**	**86,5**	**116,5**	−1,4	−0,4	1,0
01g110460	3-oxo-5-alpha-steroid 4-dehydrogenase family protein	**13,7**	**35,1**	**12,0**	**7,0**	−1,4	0,8	2,3
01g107170	Zinc finger protein	**26,9**	**66,0**	**29,5**	**39,1**	−1,3	−0,4	0,8
01g111500	MYB transcription factor	**38,4**	**92,9**	**36,8**	**67,2**	−1,3	−0,9	0,5
01g108910	At2g15890	**183,9**	**433,5**	**12,4**	**43,6**	−1,2	−1,8	3,3
01g111370	Choline transporter like family	**136,1**	**319,6**	**70,4**	**98,6**	−1,2	−0,5	1,7
01g107340	Unknown protein	**44,4**	**101,3**	**43,5**	**45,5**	−1,2	−0,1	1,2
01g110760	Unknown protein	**9,7**	**20,6**	**12,0**	**14,7**	−1,1	−0,3	0,5
01g108250	Vacuolar import and degradation protein VID27	**23,2**	**47,5**	**34,8**	**31,8**	−1,0	0,1	0,6
01g111080	Gibberellin-regulated protein 2	**12,0**	**24,5**	**13,0**	**22,2**	−1,0	−0,8	0,1
01g107180	Phototropic-responsive NPH3 family protein	**215,6**	**434,9**	**171,3**	**61,3**	−1,0	1,5	2,8

*The values in columns 3–6 are normalized fluorescence data from transcriptome microarray published by Balcke et al. (2017). ^a^log_2_ fold-change between leaves and trichomes in S. habrochaites LA1777 (column 7) and S. lycopersicum (column 8). ^b^log_2_ fold change of the expression in trichomes of S. habrochaites compared to the expression in trichomes of S. lycopersicum.*

For the QTL on chromosome VII, this selection leads to only eight genes, with diverse functional annotations, including a transcription factor, a cell wall protein, two metabolic enzymes (P450 and a ketol-acid reductoisomerase), an ATP/ADP carrier protein and a receptor kinase (see **Table 3**).

**Table 3:** Potential candidate genes from the trichome shape QTL on chromosome VII.

Solyc ID	Annotation	LA1777 leaves	LA1777 trich.	LA4024 leaves	LA4024 trich.	Leaves/trich. LA1777^a^	Leaves/trich. LA4024^a^	1777/4024^b^
07g062510	Cytochrome P450	**31,9**	**555,6**	**16,0**	**88,4**	−4,1	−2,5	2,7
07g055880	Unknown Protein	**10,3**	**49,8**	**6,7**	**5,2**	−2,3	0,4	3,3
07g056430	Glutathione *S*-transferase-like protein	**35,4**	**168,1**	**11,9**	**13,4**	−2,2	−0,2	3,7
07g053280	Ketol-acid reductoisomerase	**429,1**	**1359,6**	**447,2**	**404,6**	−1,7	0,1	1,7
07g053540	Fasciclin-like arabinogalactan protein 4	**61,7**	**194,6**	**91,3**	**102,8**	−1,7	−0,2	0,9
07g054450	Transcription factor (fragment)	**17,7**	**50,5**	**33,5**	**32,6**	−1,5	0,0	0,6
07g053830	Mitochondrial ADP/ATP carrier proteins	**53,8**	**150,7**	**50,8**	**54,5**	−1,5	−0,1	1,5
07g056270	Receptor-like protein kinase	**66,0**	**143,9**	**39,9**	**98,2**	−1,1	−1,3	0,6

*The values in columns 3–6 are normalized fluorescence data from transcriptome microarray published by Balcke et al. (2017). ^a^log_2_ fold-change between leaves and trichomes in S. habrochaites LA1777 (column 7) and S. lycopersicum (column 8). ^b^log_2_ fold change of the expression in trichomes of S. habrochaites compared to the expression in trichomes of S. lycopersicum.*

## Discussion

The shape of the glandular type VI trichomes is significantly different between wild and cultivated tomato species. In particular, the *S. habrochaites* accessions have a spherical trichome head while cultivated tomatoes like *S. lycopersicum* var. *cerasiforme* WVa106 have the shape of a four-leaf clover. To quantify this trait we designed a trichome score that reflects the presence of constrictions between the glandular cells in the cultivated tomato. We could show that the shape is a quantifiable trait. All other tested wild tomato species such as *S. pennellii*, *S. chilense* or *S. arcanum* show an intermediate trichome phenotype closer to *S. lycopersicum* than to *S. habrochaites* but clearly distinguishable from both. *S. lycopersicum* and *S. pimpinellifolium* accessions, including *S. lycopersicum* var. *cerasiforme* WVa 106 which is considered to be an admixture of *S. lycopersicum* and *S. pimpinellifolium* (Ranc et al., 2008). Notably, *S. habrochaites* is the only tomato species we could identify with this typical spherical type VI trichome shape. This raises the question of the evolution of the type VI trichomes within the tomato genus: was the spherical type VI trichome an ancestral form, which was conserved in *S. habrochaites* and lost in the other species, or on the contrary is it a specific evolution of *S. habrochaites*? *S. habrochaites*, together with species of the *S. arcanum* group is more closely related to *S. lycopersicum* than *S. peruvianum* or *S. chilense* for example (Zuriaga et al., 2009). This would support the hypothesis of a recent evolution of this trait in *S. habrochaites* rather than a loss in the other species.

We created a BC1 population of the *S. habrochaites* LA1777 and *S. lycopersicum* WVa 106 parents and could quantify the shape of the trichome heads using the trichome score. This showed that the shape of the different lines based on this score follows a normal distribution, with the parents on either ends, indicating that there are multiple genes contributing to type VI trichome shape determination. None of the backcross lines reaches a score comparable to that of *S. habrochaites*, neither do the F1 plants. As, by design, none of the backcross lines are homozygous for the *S. habrochaites* genotype in any genetic loci, a gene dosage effect might play a role in preventing the backcross lines from reaching wild type like scores. However, in the progeny of one of the BC lines, plants homozygous for the QTLs on chromosomes I and VII showed high trichome scores but still not as high as that of *S. habrochaites*. The absence of *S. habrochaites* homozygosis also prevents the detection of any recessive trait originating from the wild parent.

The extensive sequence information available for both parents allowed a mapping which is essentially only limited by the population size and the recombination rates. Although the genomes of *S. habrochaites* and *S. lycopersicum* are largely syntenic (Anderson et al., 2010; Szinay et al., 2012), there are multiple lines of evidence for the presence of chromosome rearrangements, such as inversions, deletions, or mispositioning of kinetochores as shown by the observation of synaptonemal complexes of hybrids (Anderson et al., 2010). The sequencing and detailed annotation of some specific loci of *S. habrochaites* confirmed this. For example the Cf-4 locus that confers resistance to *Cladosporium fulvum* contains an expanded set of genes encoding Leucine Rich Repeat proteins on chromosome 1 (Parniske et al., 1997) and the locus on chromosome 8 that contains the santalene/bergamotene synthase gene shows significant differences with the homologous locus from *S. lycopersicum* (Matsuba et al., 2013). Perhaps most problematic for mapping is the presence of inversions as shown in the case of the Ty2 locus, which confers resistance to the tomato yellow leaf curl virus (Wolters et al., 2015). Such inversions lead to significantly reduced recombination and therefore prevent high-resolution mapping. Because recombination in our QTL, particularly the one on chromosome 1 seems to be suppressed, we cannot exclude that the order or number of the genes in these intervals in *S. habrochaites* is different to those of *S. lycopersicum*. The QTL analysis reveals that there are several QTLs contributing to the spherical trichome head. Three of the six QTLs have an LOD score only slightly above the defined significance level of 2.5, indicating that the actual contribution of these loci to type VI trichome shape has to be taken with caution. Two closely located QTLs have been predicted on both chromosomes VII and XI. However, a higher population size and finer mapping would be needed to fully exclude the possibility of there only being one and not two QTLs.

The strongest QTL is located on chromosome I and with the current mapping encompasses about 500 annotated genes (see **Supplementary Tables S3**, **S4**). Based on RNA sequencing data of leaves and trichomes previously published (Balcke et al., 2017), we could identify 370 genes expressed in trichomes and leaves of LA1777. Of these, only 23 genes have a more than twofold higher abundance in isolated trichomes compared to whole leaves in LA1777 and with a higher expression in LA1777 trichomes than in LA4024 trichomes (**Table 2**). One of the strongest regulated genes is Solyc01g110340.2.1, coding for an endoglucanase. This cell wall modifying enzyme is a good candidate as the formation of the intercellular storage cavity, which appears to be correlated to the round shape of the type VI trichomes, is likely to involve cell wall remodeling. In addition, there are two highly regulated transcription factors of the homeodomain (Solyc01g109980.2.1) and MYC (Solyc01g111500.2.1) families as well a receptor-like kinase (Solyc01g111950.2.1). The closest *Arabidopsis* homolog of Solyc01g109980 is BLH6 (BEL-LIKE HOMEODOMAIN6), which was shown to be involved in a negative regulatory complex of secondary cell wall (Liu et al., 2014). Since the outer envelope of the trichomes is likely to play a determining role in the shape, this is a potentially interesting candidate. Solyc01g111500 encodes a MYB factor whose closest *Arabidopsis* homolog is AtMYB4 (At4g38620), a transcriptional repressor of phenolics biosynthesis. A closely related MYB homolog from maize (Zm 38) is involved in repression of anthocyanin biosynthesis (Franken et al., 1994), indicating this group of MYB factors share conserved functions in the repression of phenolic compounds. Such functions could also have an impact on the formation of the outer envelope of the trichomes by regulating its phenolic content. Another potentially interesting candidate is the gene encoding 4-hydroxy-3-methylbut-2-enyl diphosphate reductase (HDR), the last enzyme of the 2-*C*-methyl-D-erythritol 4-phosphate (MEP) pathway, which supplies isoprenyl-diphosphate precursors for terpenoids in the plastids. It is significantly more expressed in trichomes of LA1777, which is in line with the larger amounts of terpenes produced in trichomes this species. Overexpression of genes involved in the biosynthesis of methylketones in *S. habrochaites* sp. *glabratum*, which also has round type VI trichomes, has been observed by Ben-Israel et al. (2009). Thus, one possibility is that the flux of metabolites exported into the cavity contributes to the shape of the trichome. However, unlike deoxy-xylulose phosphate synthase (DXS), HDR is not known to be a regulatory bottleneck in the flux through the MEP pathway. Also, Ben-Israel et al. (2009) proposed another explanation, namely that the biosynthesis of MKs, which are fatty acid derivatives, diverts a pool of fatty acid acids away from the very long chain fatty acids that are incorporated in the cuticle, thereby influencing the shape of the trichome. In the case of *S. habrochaites* LA1777, this is unlikely to be the case because sesquiterpenoids are the major products of type VI trichomes and therefore should not alter the composition of the cuticle. Another potentially interesting candidate is Solyc01g108670.2.1, which encodes a protein of the phragmoplast-associated kinesin family. In *Arabidopsis* there are two homologs of this type of kinesin, PHRAGMOPLAST ORIENTING KINESIN 1 and 2, whose combined mutation leads to defects in the orientation of the cell division plane (Lipka et al., 2014).

With the same criteria, only seven genes from the QTL on chromosome VII could be retained (**Table 3**). There is one transcription factor (Solyc07g054450.2.1) with similarity to ULTRAPETALA1, a repressor of inflorescence and flower meristem size, a receptor like kinase (Solyc07g056270.2.1), a fasciclin-like arabinogalactan protein (Solyc07g053540.1.1) and a protein of unknown function (Solyc07g055880.1.1). The gene with the strongest differential expression (Solyc07g062510.2.1) encodes a cytochrome P450 oxidase of the CYP72 family, whose function is, however, unclear. Also of note in this list is the presence of a ketol-acid reductoisomerase, an enzyme involved in branched chain amino acid and in pantothenate and coenzyme A biosynthesis. How these genes could contribute in any way, if any, to type VI trichome shape is unclear and will need further functional analysis.

Furthermore, because the trichome preparation used for our transcriptome data is made up of mature trichomes and the shape of the trichomes is a developmental trait, it is possible that we are missing genes that are only expressed in a specific stage of trichome development. We recently described a method to isolate such young trichomes (Bergau et al., 2016). Transcriptome data from these developing trichomes is being produced and will be used to mine for differentially expressed genes that are located in the QTLs identified in this study.

Identification of the causal gene(s) will require a combination of refined mapping, use of developmental stage-specific transcriptomics, and functional testing of the genes, for example by virus-induced gene silencing as shown by (Besser et al., 2009) or by gene inactivation with CRISPR/Cas9 (Ito et al., 2015).

All QTLs detected here contribute in the same direction and the additive effects toward a rounder trichome head are driven by the presence of the *S. habrochaites* alleles within the QTLs. This is mirrored by the fact that *S. habrochaites* lines show the highest and *S. lycopersicum* the lowest trichome scores. We could not detect strong epistasis effects regulating the QTLs showing the contributors are independent from each other. These results suggest that the type VI trichome phenotype is mostly contributed by the dominant expression of genes from the *S. habrochaites* genome and that these different genes are not part of a unique pathway but individually contribute to this phenotype.

The effect of the QTL on chromosome I could be confirmed by genotyping the self-pollinated progeny of two backcross lines. A BC1 line with a low trichome score and without *S. habrochaites* genetic information within the QTL produced offspring with a low score, while a BC1 line that was heterozygous for *S. habrochaites* markers spanning the chromosome I QTL produced offspring with a further segregating trichome phenotype with the top-scoring plants retaining the *S. habrochaites* genotype on Chromosome I and the bottom-scoring plants losing it. Even in plants that were homozygous for *S. habrochaites* markers within the QTL, the trichome score did not reach *S. habrochaites* levels, confirming that there are additional contributing factors.

One of the factors might be the accumulation of sesquiterpenoids derived from α-santalene and various forms of bergamotene. While *S. lycopersicum* does not make these compounds but instead essentially monoterpenes, they are accumulating to high amounts (over 10 mg/g leaf fresh weight) in *S. habrochaites* LA1777 (Coates et al., 1988). The enzymes needed to synthesize santalene and bergamotene in LA1777 for example are located in a cluster on chromosome VIII (Sallaud et al., 2009), close to the weak QTL identified on that chromosome. Furthermore, lines in the backcross population accumulating santalene have a slightly higher trichome score compared to lines that do not accumulate the sesquiterpene. The accumulation of these secondary metabolites within the trichome heads could lead to an increased pressure thereby influencing the trichome shape and thereby the trichome score we measure. A similar hypothesis was proposed in a previous study investigating the correlation between the type VI trichome shape and genes involved in the biosynthesis of methylketones in *S. habrochaites* ssp. *glabratum* (Ben-Israel et al., 2009). It should be noted, however, that this does not appear to be a major contributing factor and that the major QTLs we identified on chromosome I and VII do not contain genes that are involved in the biosynthesis of these sesquiterpenoids.

Our findings show that the shape of glandular trichomes in tomato is a quantifiable trait. The genes behind these morphological differences remain elusive, but additional transcriptomics data from developing trichomes combined with medium-throughput VIGS or targeted deletions of sub-intervals of the QTL with genome-editing tools should help us identify the causal genes. This should provide crucial data to breed trichome-based insect resistance traits in cultivated tomato.

## Author Contributions

SB performed all the experiments except the fluorescence microscopy. NB performed the fluorescence microscopy. AT conceived the project and contributed to the experimental design. SB and AT analyzed the results and wrote the manuscript. All authors read and approved the manuscript before submission.

## Conflict of Interest Statement

The authors declare that the research was conducted in the absence of any commercial or financial relationships that could be construed as a potential conflict of interest.

## Footnotes

1 https://imagej.net/Welcome

## Supplementary Material

FIGURE S1 Fluorescence microscopy of isolated type VI trichomes from *Solanum habrochaites* LA1777 *and S. lycopersicum* WVa106.

FIGURE S2 Examples of type VI trichome shape from several wild tomato species, other than *S. habrochaites*.

FIGURE S3 Correlation between trichome score and size of the intercellular storage cavity. Type VI trichomes images were taken with a laser-scanning confocal microscope (see section “Materials and Methods”) at the z-position where the area of the cavity is the largest. The scale bar represents 20 μm. These images were used to calculate the trichome score (see **Figure 1**) and the area of the cavity. Ten images were taken per line, except for Wva106 where three images were taken. The area of the cavity was then normalized to the total area of the trichome. The data were then plotted and curve-fitted in Sigma Plot version 10. The best fit was obtained with a second degree polynomial curve (see equation on the graph) with a *r*^2^ of 0.93. The dashed line represents the fitted curve. The solid lines represent the curves for a 99% confidence interval.

FIGURE S4 Linkage map with the relative (on the left; in cM) and absolute (on the right; in MB) positioning of the markers.

TABLE S1 Molecular markers used in this study.

TABLE S2 Mapping data.

TABLE S3 Genes of the QTL on chromosome I.

TABLE S4 Examples of polymorphisms found in 5 genes randomly selected in the QTL of chromosome 1.
