# Supplementary material for: QTL Mapping of the Shape of Type VI Glandular Trichomes in Tomato
Source: Front Plant Sci. 2018 Sep 26;9:1421. doi: 10.3389/fpls.2018.01421 (PMC6168718; doi:10.3389/fpls.2018.01421)
Supplement: FIGURE S1 — Fluorescence microscopy of isolated type VI trichomes from Solanum habrochaites LA1777 and S. lycopersicum WVa106. [file Presentation_1.pptx]

## Slide 1
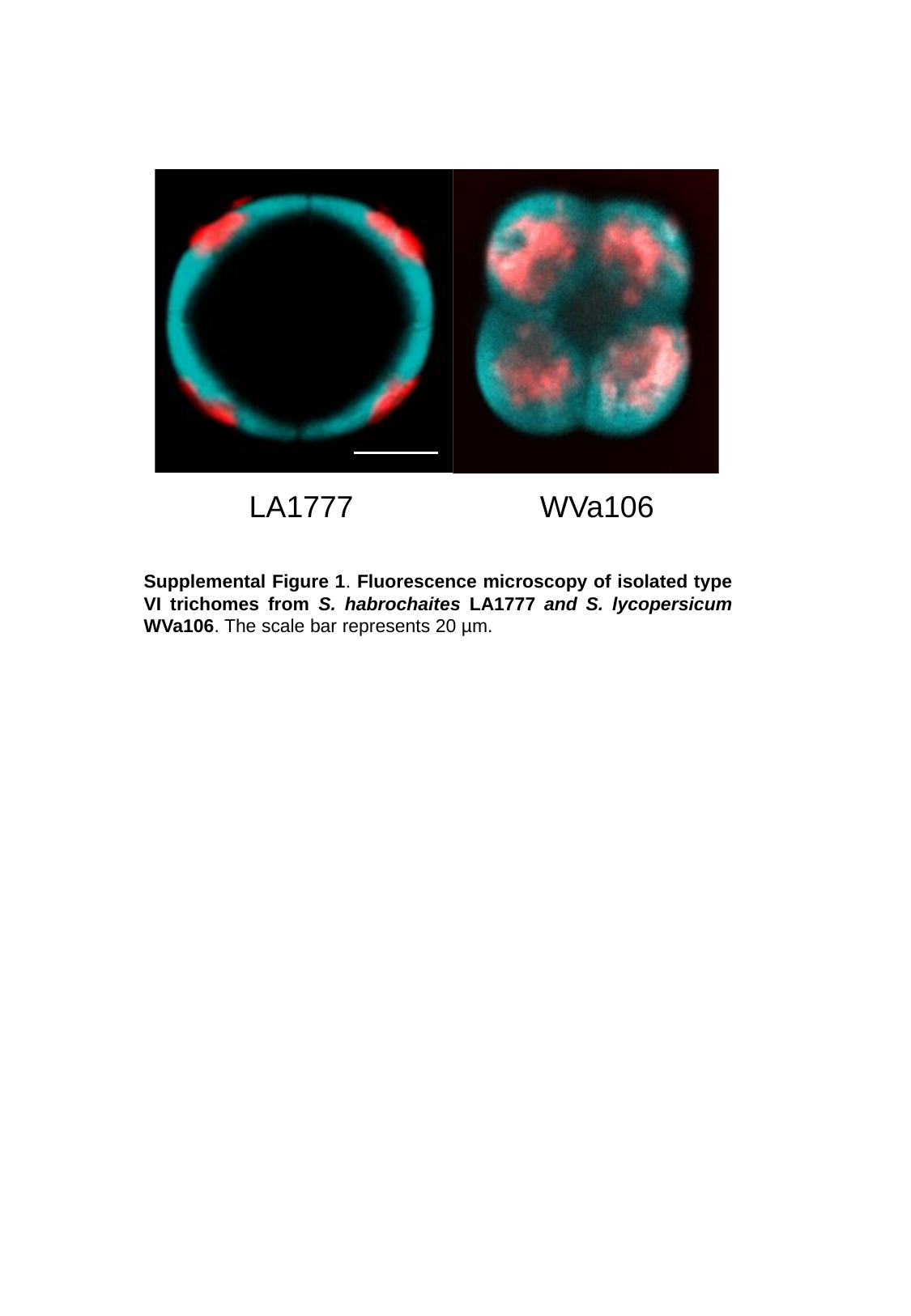

LA1777
WVa106
Supplemental Figure 1. Fluorescence microscopy of isolated type VI trichomes from S. habrochaites LA1777 and S. lycopersicum WVa106. The scale bar represents 20 µm.

## Slide 2
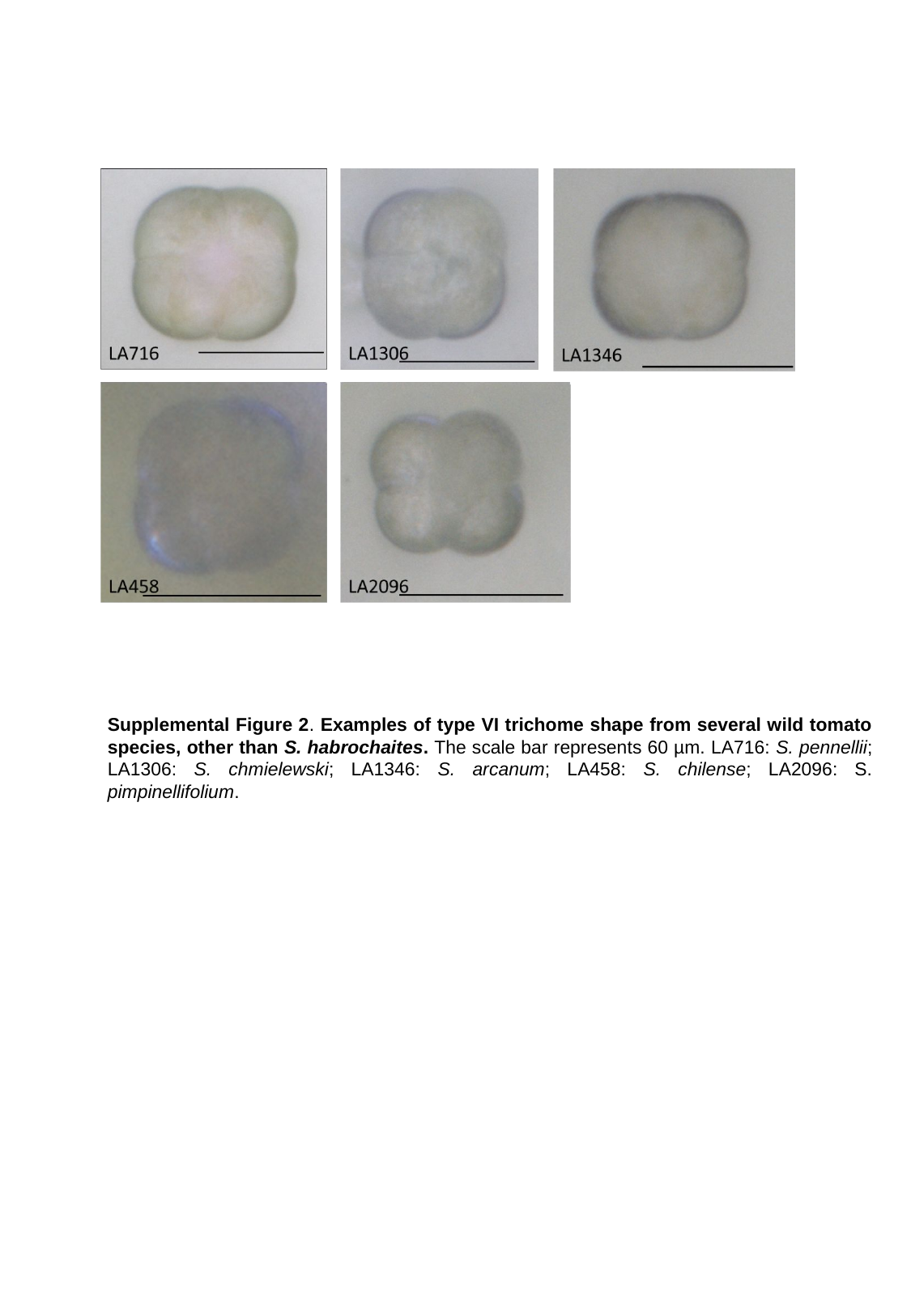

Supplemental Figure 2. Examples of type VI trichome shape from several wild tomato species, other than S. habrochaites. The scale bar represents 60 µm. LA716: S. pennellii; LA1306: S. chmielewski; LA1346: S. arcanum; LA458: S. chilense; LA2096: S. pimpinellifolium.

## Slide 3
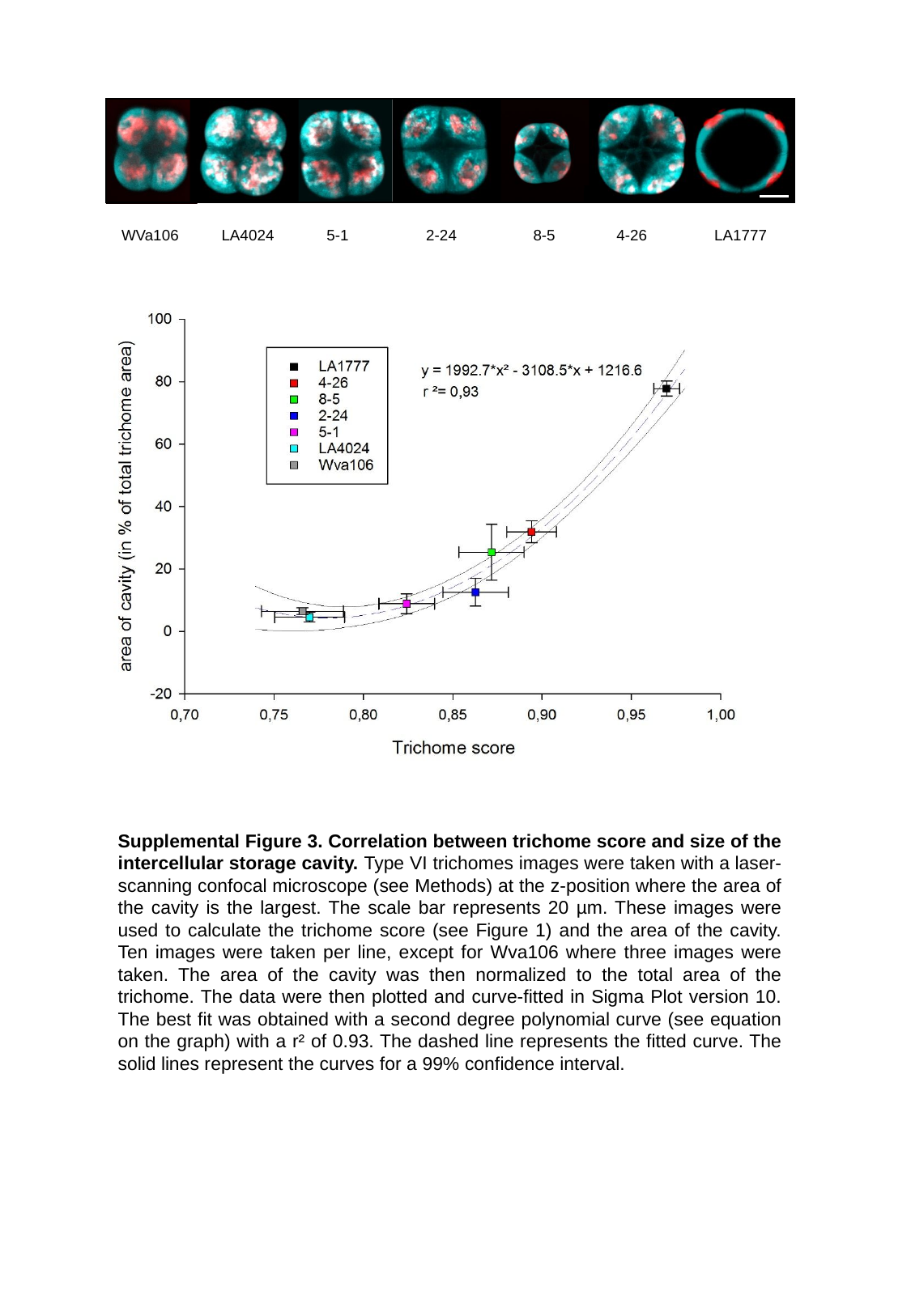

WVa106
LA4024
5-1
2-24
8-5
4-26
LA1777
20 um
Supplemental Figure 3. Correlation between trichome score and size of the intercellular storage cavity. Type VI trichomes images were taken with a laser-scanning confocal microscope (see Methods) at the z-position where the area of the cavity is the largest. The scale bar represents 20 µm. These images were used to calculate the trichome score (see Figure 1) and the area of the cavity. Ten images were taken per line, except for Wva106 where three images were taken. The area of the cavity was then normalized to the total area of the trichome. The data were then plotted and curve-fitted in Sigma Plot version 10. The best fit was obtained with a second degree polynomial curve (see equation on the graph) with a r² of 0.93. The dashed line represents the fitted curve. The solid lines represent the curves for a 99% confidence interval.

## Slide 4
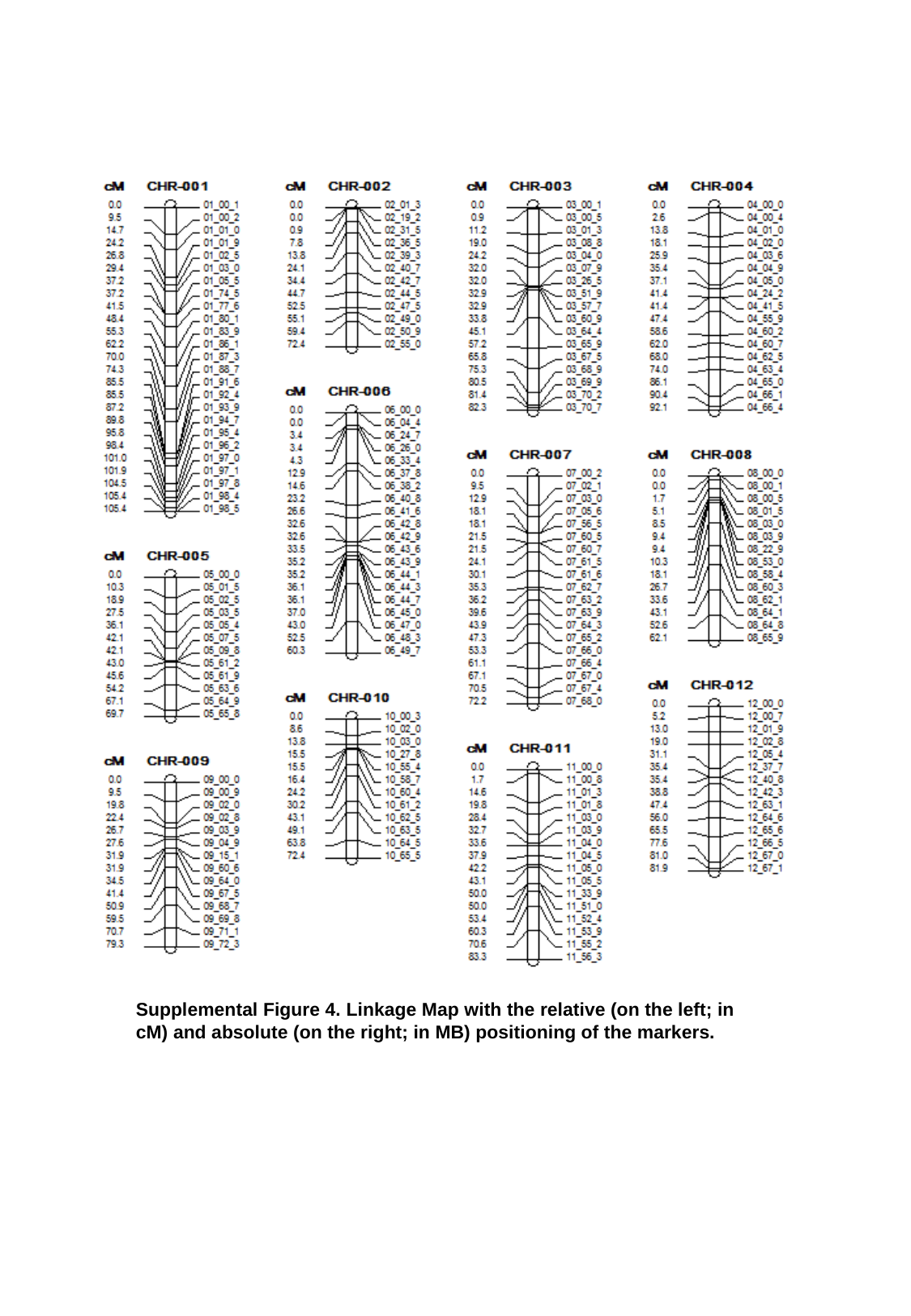

Supplemental Figure 4. Linkage Map with the relative (on the left; in cM) and absolute (on the right; in MB) positioning of the markers.
